# Stable Bacterial Cores and Patchy Eukaryotic Communities Shape the Summer Microbiomes of Patagonian Fjord Ecosystems Across Spatial, Functional and Temporal Gradients

**DOI:** 10.1111/1758-2229.70412

**Published:** 2026-09-08

**Authors:** Nicole Trefault, Susana Rodríguez‐Marconi, María Fernanda Manrique‐de‐la‐Cuba, Patricio Flores‐Herrera, Clara Natalia Rodríguez‐Flórez, Bernd Krock

**Affiliations:** ^1^ Centro GEMA‐Genómica, Ecología y Medio Ambiente Universidad Mayor Santiago Chile; ^2^ Millenium Nucleus in Marine Agronomy of Seaweed Holobionts (MASH) Puerto Montt Chile; ^3^ FONDAP Center IDEAL‐ Dynamics of High Latitude Marine Ecosystem Valdivia Chile; ^4^ Secretariat of Environment and Sustainable Development of Boyacá Concepción Colombia; ^5^ Alfred Wegener Institut‐Helmholtz Zentrum für Polar‐und Meeresforschung Bremerhaven Germany; ^6^ Centro de Investigación Oceanográfica COPAS COASTAL Universidad de Concepción Concepción Chile

**Keywords:** core microbiome, glacier runoff, microbial communities, microbial interaction networks, Patagonian fjords, salinity gradients

## Abstract

Patagonian fjord ecosystems are shaped by a combination of glacial, oceanographic and climatic factors that generate physical and chemical gradients. In these environments, microbial communities play essential roles in ecosystem functioning, with temporal and depth‐related compositional shifts often linked to glacial meltwater input. In this study, we characterised microbial communities from southern Patagonia and the Drake Passage using high‐throughput sequencing of the 16S and 18S rRNA genes across seven study areas. We found that bacterial and eukaryotic communities differed across the sampling areas, with salinity showing the strongest association with community composition in both microbial domains. Bacterial core communities, defined here using a combined occupancy–abundance approach, were widespread throughout fjords and channels, whereas eukaryotic core communities were spatially restricted. Network analyses revealed contrasting modes of community organisation across these fjord systems, with land‐terminating glaciers displaying more hub microorganisms (highly connected bacteria and microbial eukaryotes) than water‐terminating glaciers. Short‐term analyses identified microbial taxa that varied significantly in abundance over a 10‐day period and were present in inter‐domain co‐occurrence networks. Together, these results identify a small set of abundant and well‐connected microorganisms that structure summer microbiomes in Patagonian fjords and may link glacial runoff regimes into distinct modes of community interactions.

## Graphical abstract

Our study shows that microbial community diversity in Patagonian fjords varies with glacial influence and salinity, with a widespread bacterial core but a patchier eukaryotic core. Distinct co‐occurrence networks and marked short‐term community shifts occur in water‑ versus land‐terminating glacier fjords, underscoring meltwater influence in this marine ecosystem.

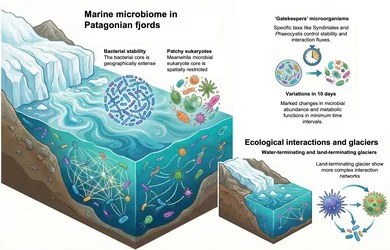

## 1 Introduction

Patagonian fjord ecosystems, including several bays, estuaries, gulfs, islands and channels, are among the largest fjord systems globally and represent a dynamic interface between terrestrial, freshwater and marine environments. These systems are shaped by a unique combination of glacial, oceanographic and climatic forces, creating sharp physical and chemical gradients that support high primary productivity and microbial diversity (Iriarte et al. 2010; González et al. 2013).

Two main features—riverine discharges and nutrient inputs from oceanic waters—modulate the size structure and seasonal succession of phytoplankton communities across Patagonian fjords (Cuevas et al. 2019). In southern Patagonia, low concentrations of silicic acid favour non‐siliceous flagellates and other non‐siliceous phytoplankton species and the community structure is mainly dominated by small‐sized fractions (i.e., nano‐ and pico‐phytoplankton), which could indicate lower carbon transfer and export efficiency, as well as vigorous recycling of organic matter through the microbial loop (Iriarte et al. 2017). On the other side, in specific locations such as the Beagle Channel, solar radiation is considered the primary driver of phytoplankton variation throughout the year (Almandoz et al. 2011). Seasonal phytoplankton blooms have been documented beginning in austral spring (October) with diatom dominance, followed by increasing abundances of dinoflagellates, cryptophytes and haptophytes in summer (Almandoz et al. 2011).

Microbial communities are key players in nutrient cycling, organic matter degradation and overall ecosystem functioning in fjord environments. Several studies have reported pronounced temporal, seasonal and depth‐related shifts in microbial community composition, often linked to glacial meltwater input, freshwater stratification and organic matter availability (Delpech et al. 2021; Gutiérrez et al. 2015). In addition to temporal variability, spatial heterogeneity is a common feature of fjord ecosystems. Microbial community structures are influenced by sharp gradients in salinity, temperature, turbidity and nutrient regimes. In the Baker Channel (northern Patagonia), bacterial communities show clear spatial separation along estuarine salinity gradients (Tamayo‐Leiva et al. 2021). Moreover, different active microbial communities were found in two fjords with contrasting morphological and hydrological features: Pia, a marine‐terminating glacier and Yendegaia, a land‐terminating glacier (Maturana‐Martínez et al. 2021). However, it is unknown whether the patterns of bacterial differentiation between land‐terminating and water‐terminating glaciers also apply to the whole microbial community, including eukaryotes and their ecological interactions.

One of the most prominent channels in southern Patagonia is the Beagle Channel, an interoceanic passage located at the southern tip of South America. It connects the Pacific and Atlantic oceans through a complex system of fjords and glacier‐influenced coastal zones. The water column is generally well‐mixed, primarily shaped by atmospheric forcing and influenced by the interaction between Subantarctic Surface Water and Estuarine Water of continental origin (Giesecke et al. 2021). Adjacent to the Beagle Channel is the Garibaldi Fjord, part of the Alberto de Agostini National Park in Chile. This area is a UNESCO Biosphere Reserve and includes the Garibaldi Glacier, characterised by a medial moraine, meaning it is the union of two separate ice flows (Bown et al. 2014).

Recent work has shown that the ecological importance of fjord microbial communities in southern Patagonia is high and that these communities are highly dynamic across short spatial and temporal scales, with variability often driven more by biological interactions and local hydrographic conditions than by physical gradients (Malits et al. 2023; Carrasco et al. 2023; Cadaillon et al. 2024).

Despite these advances, the mechanisms linking regional connectivity, local environmental filtering and the persistence of core microbial taxa across this ecosystem remain to be studied. In this context, identifying a core community is essential to understanding which microorganisms persist across environmental gradients, such as those in glacier runoff. The core microbiome concept refers to any set of microbial taxa, as well as the associated genomic or functional attributes characteristic of a specific host or environment (Turnbaugh et al. 2007; Hamady and Knight 2009; Shade and Handelsman 2012). Such recurrent taxa seem to represent the most ecologically and functionally important microbial associates of that host or environment under the conditions sampled (Neu et al. 2021) and has been shown to be a useful tool to describe persistent microbial populations in marine environments (Mende et al. 2019; Krabberød et al. 2022).

Microbial community composition in fjords is not determined by environmental filtering alone but also emerges from ecological interactions among bacteria, microbial eukaryotes and higher trophic levels, which are tightly coupled through biogeochemical cycling and marine food‐web linkages (Deutschmann et al. 2024). Co‐occurrence network analyses of amplicon or metagenomic data have become powerful tools for inferring putative ecological associations, identifying highly connected nodes and screening for candidate keystone taxa that may stabilise or reorganise community structure (Steele et al. 2011; Banerjee et al. 2018; Deutschmann et al. 2024).

Here, we aim to understand the influence of glacier runoff in the fjords of southern Patagonia on the community structure and functionality of bacterial and microbial eukaryote assemblages. Our main objectives were: (1) to spatially characterise the bacterial and microbial eukaryotes, including their core communities; (2) to functionally and trophically differentiate bacterial and microbial eukaryote communities across fjord and channel systems of southern Patagonia; (3) to evaluate microbial interactions between these two microbial components, differentiating between water‐terminating and land‐terminating glaciers; and (4) to follow a short‐scale temporal dynamic in the taxonomic and functional diversity and composition of the bacterial and microbial eukaryote communities in the Garibaldi Fjord.

## 2 Experimental Procedures

### 2.1 Sampling

**FIGURE 1:**
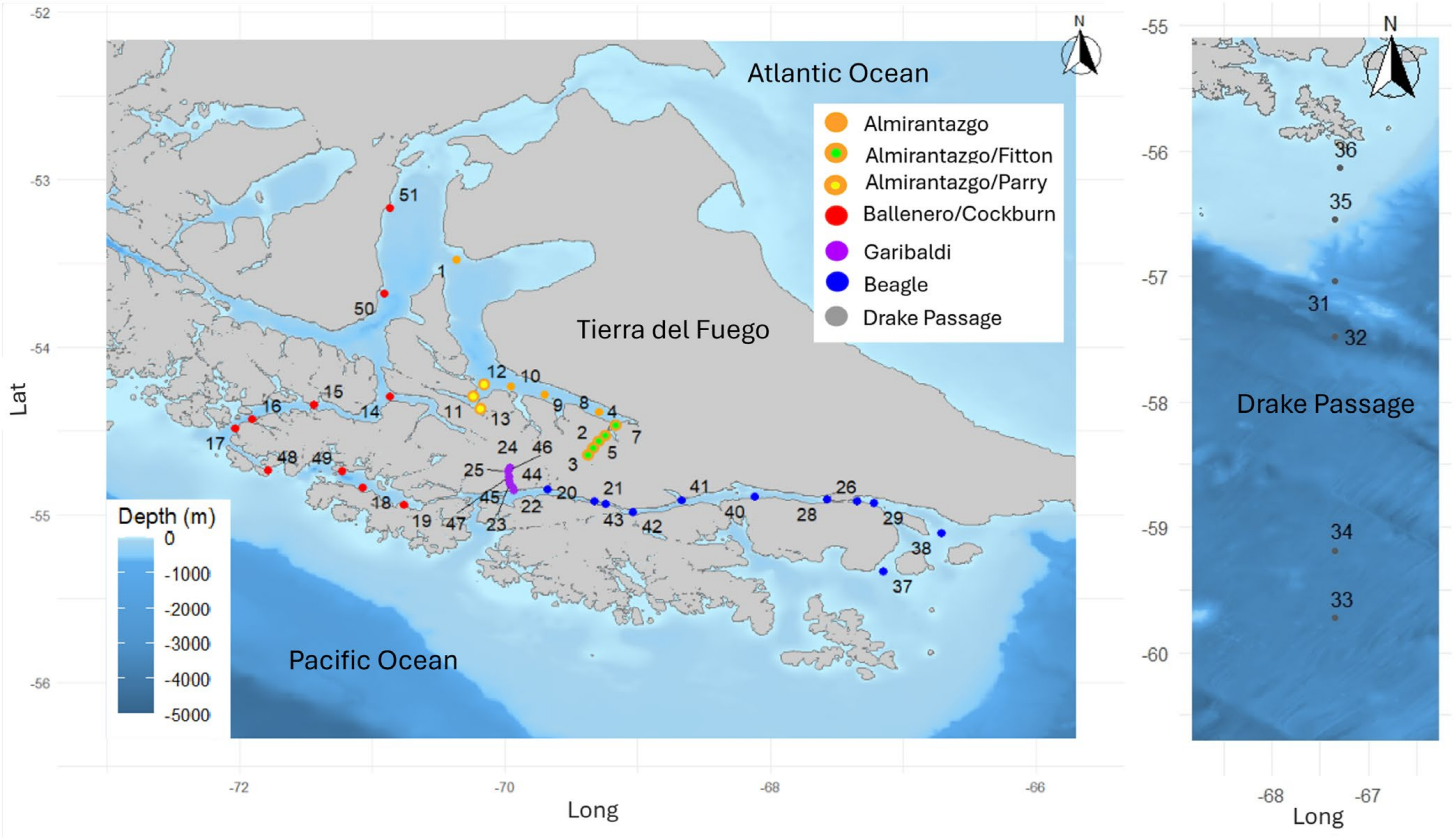
Maps of the study areas indicating sampling stations during the FjordFlux cruise and the seven study areas defined for specific analysis in the Patagonian fjords and in the Drake Passage. (Left) Areas in the southern Patagonian fjord system: (1) Almirantazgo Sound, (2) Fitton Bay, (3) Parry Fjord, (4) Cockburn/Ballenero Channels, (5) Garibaldi Fjord and (6) Beagle Channel. (Right) Area with the transect to open waters in the Drake Passage.

We sampled microbial communities during the FjordFlux (M179/2) cruise aboard the R/V Meteor from January 15th to February 14th, 2022. This cruise explored the central part of the Magellan Strait, including Almirantazgo Sound, with a special focus on Parry Fjord, which features a water‐terminating glacier and Fitton Bay, which has a land‐terminating glacier. The expedition continued through the Gabriel Channel and headed towards the Pacific via Cockburn Channel, followed by a short coastal transect eastward before entering Ballenero Channel. Extensive work was done in the Beagle Channel, with special focus on Garibaldi Fjord and its water‐terminating glacier. The transect continued eastward to the Atlantic mouth of the Beagle Channel and, to compare the coastal internal waters of the Tierra del Fuego archipelago with open ocean waters, a southward transect was made to almost 60° S (Figure 1).

We sampled 48 stations across seven target areas: Almirantazgo area with 14 stations; Garibaldi Fjord with seven; Cockburn/Ballenero Channels with 10; Beagle Channel with 11; and Drake Passage with six (Table S1), using a CTD rosette equipped with 24 Niskin bottles of 12 L. The Almirantazgo area was subdivided into three areas: Almirantazgo Sound (four stations), Parry Fjord (seven) and Fitton Bay (three) (Figure 1). At each station, 5 L of seawater samples were collected at depths above 30 m (details of geographic locations, depths and sample types are provided in Table S1). For DNA extraction, subsamples were size fractionated through 0.2 μm (GSWP) and 3 μm (SSWP) diameter pore Millipore filters using Swinnex holder systems (47 mm) and a Cole Parmer 1–100 rpm peristaltic pump.

Hydrographic profiles were obtained using an SBE 911plus CTD unit (Sea‐Bird Electronics Inc.) mounted on an SBE 32 Carousel Water Sampler fitted with 24 Ocean Test Equipment Inc. bottles (20 L each). Instrumentation included duplicate temperature (SBE 3) and conductivity (SBE 4) sensors, a Digiquartz pressure sensor, an Aanderaa Optode 4831F oxygen sensor, a Benthos altimeter and a combined chlorophyll fluorometer/turbidity sensor (WET Labs ECO_AFL FL), all factory pre‐calibrated. Data acquisition used Seasave V 7.23.2 software, with subsequent processing performed in Sea‐Bird SBE Data Processing.

Flow cytometry samples (1.5 mL) were fixed in triplicate using Glutaraldehyde/pluronic acid (0.25%/0.1% final concentration) for microbial abundance determination. Samples were fixed for 20 min at room temperature, flash‐frozen in liquid nitrogen and stored at −80°C until further analysis.

For nutrient analysis, unfiltered seawater (nitrate, nitrite, ammonium, phosphate and silicate) was collected in 50 mL LDPE bottles and processed following the protocol of Graeve et al. (2018) and consistent with our previous report in Rodríguez‐Marconi et al. (2024). Chlorophyll‐a (Chl‐a) samples (up to 3 L per station) were collected onto pre‐washed glass fibre filters (47 mm diameter, ~0.7 μm pore size; Whatman, UK) and rinsed with 0.2 μm‐filtered seawater. Filters were stored frozen at −80°C aboard the vessel and processed for Chl‐a analysis in the laboratory after the cruise.

### 2.2 Flow Cytometry, Nutrient Analysis and Chlorophyll a Quantification

Cell abundances of total bacteria, photosynthetic picoeukaryotes (PPE), photosynthetic nanoeukaryotes (PNE) and Cryptophytes (CRY) were determined using an Accuri C6 Plus flow cytometer (Becton Dickinson). Light scatter and fluorescence were normalised by adding 1 and 3 μm fluorescent beads. For bacterium quantification, samples were stained with Sybr Green I and counts were based on side scatter and green fluorescence (530/40 nm). To identify phytoplankton groups, we used a 488 nm excitation laser and collected fluorescence at 580/15 nm (orange fluorescence) as a proxy for phycoerythrin and at 670/LP (red fluorescence) as a proxy for chlorophyll, as described by Marie et al. (2005).

For nutrient analyses, unfiltered water (nitrate, nitrite, ammonium, phosphate and silicate) was collected in 50 mL LDPE bottles and analysed as described in Graeve et al. (2018). To determine Chl‐a concentration, pigment extraction was performed using a 90% acetone‐water solution with overnight incubation at 4°C. The extracts were then centrifuged and the supernatant fluorescence was measured at 665 nm using a pre‐calibrated TD‐700 laboratory fluorometer (Turner Designs, San Jose, USA). Chl‐a concentration was calculated following EPA method 445 (Arar and Collins 1997).

### 2.3 DNA Extraction and Amplicon Sequencing

DNA was extracted following the CTAB‐based protocol previously applied to polar fjord samples in Rodríguez‐Marconi et al. (2024). Filters were thawed under sterile conditions and half of each filter was cut into 1 mm^2^ fragments and lysed for 1 h at 37°C in TE 1×/0.15 M NaCl buffer supplemented with 50 μL of 10% SDS and 4 μL of 20 mg mL^−1^ proteinase K. Lysates were combined with 100 μL of 5 M NaCl and 80 μL of CTAB extraction buffer (10% CTAB, 0.7% NaCl) and incubated for 10 min at 65°C, after which proteins were removed by phenol‐chloroform extraction (Doyle and Doyle 1987). DNA was precipitated using isopropyl alcohol at −20°C for 1 h and resuspended in 40 μL deionised water (Milli‐Q, Millipore). DNA integrity was evaluated by agarose gel electrophoresis and quantified using a fluorometric assay (Qubit 2.0 fluorometer).

For analyses of the bacterial communities, the hypervariable region V4 of the 16S rRNA gene was amplified using B969F and BA1406R primer pairs; and for analyses of the microbial eukaryotic communities, the hypervariable region V4 of the 18S rRNA gene was amplified using E572F and E1009R primer pairs (Comeau et al. 2011). Amplification, library preparation and sequencing were carried out at the IMR sequencing facility (https://imr.bio/index.html) using an Illumina MiSeq i100 platform with 2 × 300 bp paired‐end reads and XLEAP‐SBS chemistry.

### 2.4 Amplicon Sequencing Data Analysis

Sequences were processed on a per‐run basis for Amplicon Sequence Variant (ASV) inference using DADA2 package version 1.20.068 within the R environment version 4.1.3 (R Core Team 2021). For community composition, alpha and beta diversity, and functional group classification analyses, dinoflagellate sequences were separated from other eukaryotes, considering that dinoflagellate genomes contain large numbers of RNA operons (Lin 2011), which could lead to an overestimation of their actual abundance when compared with other plankton groups. Diversity indices were calculated in the web tool MicrobiomeAnalyst (Dhariwal et al. 2017) using default settings. Alpha diversity normality tests and paired comparisons were performed in Past 4.13 (Hammer et al. 2001). Beta diversity was evaluated using the Bray–Curtis dissimilarity index to quantify differences in community composition among sampling areas. Prior to analysis, ASV count tables were transformed to relative abundances. Community composition dissimilarities among sampling areas were explored using Non‐metric Multidimensional Scaling (NMDS) based on Bray–Curtis distances. NMDS was performed using the metaMDS function from the vegan package (v. 2.6–4) in R (Oksanen et al. 2022), with two dimensions (*k* = 2) and 100 random starts to ensure convergence to a stable ordination solution. To avoid potential biases originating from temporal variability, stations on the return track (37–51) were excluded from diversity analysis.

Sample grouping by area was visually represented using ellipses computed with the geom_mark_ellipse function from ggforce (Pedersen 2025). Each ellipse corresponds to the covariance structure of the points belonging to a given area and is drawn assuming an approximately elliptical distribution of samples in the NMDS space. Differences in community composition among areas were tested using permutational multivariate analysis of variance (PERMANOVA) with 999 permutations (adonis2, vegan), followed by pairwise PERMANOVAs adjusted for multiple comparisons using the false discovery rate (FDR) method.

Redundancy analysis (RDA) was performed to explore the relationship between microbial community composition and environmental variables. ASVs were rarefied to account for differences in sequencing depth. ASV counts were Hellinger‐transformed to reduce the influence of highly abundant taxa. Environmental variables were cleaned by removing columns with > 50% missing values and imputing remaining missing values with the column median. Variables with zero variance were excluded. All environmental variables were standardised (*z*‐score) before RDA. RDA was performed using the vegan package (Oksanen et al. 2022) in R. Forward selection based on permutation tests (999 permutations) was applied to identify the subset of environmental variables that significantly explained variation in ASV composition. The proportion of variance explained by the model was quantified using the adjusted *R* ^2^. Significance of the overall model, individual axes and explanatory variables was assessed with permutation ANOVA. Visualisation of RDA results was performed using ggplot2.

Chl‐a, nutrients, alpha diversity and community composition figures were constructed in GraphPad Prism 8.01 for Windows.

### 2.5 Identification of Core Microbiomes

To evaluate the ubiquity, dominance and persistence of microbial ASVs, that is, core, variable and infrequent communities, a relative abundance threshold of 0.1% was set for each ASV. ASVs shared by at least 80% of samples were designated as core microbiome, ASVs shared by 10 to 79.99% of the samples were called variable community and ASVs present in less than 10% of the samples were called infrequent (i.e., uncommon) community. For this analysis, dinoflagellate and microbial eukaryote sequences were not separated, as these data were transformed to absence/presence, reducing the bias originated by differences in 18S rRNA copy number.

### 2.6 Functional Profiling for Bacteria

The functional profiles of microbial communities were inferred using the Tax4Fun pipeline and relative abundances of KEGG pathways (Kanehisa et al. 2016) were used for comparative analyses among sampling areas. The table containing pathway abundances was imported into R (v4.3.0) and processed with the *tidyverse* package (Wickham et al. 2019). Relative abundances were standardised within each pathway by calculating *Z*‐scores, defined as the deviation of each sample's abundance from the mean across all samples, divided by the standard deviation. This normalisation allows direct comparison of pathways with different baseline abundance levels. For visualisation, *Z*‐scores were averaged by area and represented as a heatmap using *ggplot2* (Wickham 2016), with blue and red colours indicating below‐ and above‐average abundances, respectively. Statistical differences in pathway abundances among areas were assessed using Kruskal–Wallis tests followed by Benjamini–Hochberg correction for multiple comparisons.

Functions were predicted up to KEGG Pathway Hierarchy Level 3, corresponding to 326 KEGG Orthologs distributed across five categories: cellular processes, environmental information processing, genetic information processing, organismal systems and metabolism.

### 2.7 Classification of Functional Groups in Microbial Eukaryotes

After data preprocessing and aggregation at the genus level, genera within both the non‐dinoflagellate and dinoflagellate communities were categorised into three trophic groups: phytoplankton, protozooplankton, mixoplankton, based on the work of Schneider et al. (2020).

To assign the taxonomy at the species level in cases where the automatic assignment was not available, ASVs were annotated with the highest score against NCBI 18S rRNA and nt/nr databases (16‐03‐2024), using BLASTN 2.15.0+ https://paperpile.com/c/ApC9P2/PGwm (Altschul et al. 1990), provided they agreed with the PR^2^ taxonomy initially assigned. The trophic and lifestyle group categorisation was complemented with annotations of species/genus‐specific information from other studies (Gran‐Stadniczeñko et al. 2019; Xiong et al. 2021; Genitsaris et al. 2022; Lapeyra Martin et al. 2022, https://paperpile.com/c/ApC9P2/4xVN+neTl+f4Rt+R8Sy).

### 2.8 Co‐Occurrence Networks

We selected only samples with reads available for both 16S rRNA and 18S rRNA from glacier‐influenced coastal zones: Parry Bay, Fitton Bay and Garibaldi Bay. Fractions of pico‐ and nanoplankton from the same sample were merged using phyloseq (McMurdie and Holmes 2013) in R. Samples were subset by zone. After this, absolute abundances were rarefied to the sample with the lowest sequence count by each zone. ASVs with less than three counts by sample were removed. We used the resulting ASV and taxonomy tables from 16S and 18S rRNA by zone to infer bipartite co‐occurrence networks. Inference was performed using the CoNet plugin version 1.1.1.beta (Faust and Raes 2016) of Cytoscape version 3.9.1 (Shannon et al. 2003). We discarded ASVs present in less than 90% of the samples. Pairwise correlations between 16S and 18S rRNA matrices were calculated by combining the Pearson and Spearman measures and then requesting the network intersection using scoremethodnum. Correlation coefficient thresholds were set to 0.8 for both positive and negative values. To permit correlations, we set a threshold of 90% for the minimum ASV occurrence and non‐double‐zero value pairs across samples. The *p*‐value was set to 0.01 using the Benjamini–Hochberg correction and Brown's method was used to combine the networks from each measure. Randomisation was set with 1000 iterations. The resulting networks were visualised with the Bipartite Layout and Organic Layout, implemented within Cytoscape. The topological properties of each network were calculated using the NetworkAnalizer plugin. Node‐level topological values were imported into R to perform statistical comparisons between networks using pairwise Wilcoxon tests with Benjamini–Hochberg (BH) correction.

In Cytoscape, we subset each network to the 25 nodes with the highest betweenness centrality (i.e., nodes that frequently appear on the shortest paths between other pairs of nodes) to reveal critical connector nodes that may regulate interactions and information flow among other nodes and likely concentrate significant ecological relationships. Unconnected nodes in subsets (i.e., isolated bridge nodes resulting from selection) were hidden from the visualisation.

Critical connector nodes were compared with core members of each area to evaluate possible key members of the microbial community.

## 3 Results and Discussion

### 3.1 Freshwater Inputs Drive Distributions of Nutrients and Microbial Cell Abundance in the Southern Patagonian Fjords and Channels

In Almirantazgo Sound (see Figure 1), surface waters were warmer and less saline than deeper waters (> 10 m), consistent with glacial meltwater input from Parry Fjord (Figure S1). Freshwater influence extended throughout the fjord–channel system. Surface temperature declined poleward, from 11°C in Almirantazgo to 6°C in the South Drake Passage (Figure S2). Surface nitrate and phosphate concentrations were lower in fjords and channels than in the Drake Passage, while silicic acid peaked near freshwater inputs at Parry and Garibaldi fjords (stations 5 and 22; Figure S3). These conditions likely result from terrestrial–marine coupling, where glacial meltwater dilutes surface salinity, delivers nutrients associated with terrigenous material and increases water‐column stability (Iriarte et al. 2014; Cuevas et al. 2019; Tamayo‐Leiva et al. 2021).

Eukaryotic microbial cells were concentrated in the upper 30 m, except in the Drake Passage, where they extended to 100 m (Figure S3). In the fjords and channels, strong surface stratification and high turbidity associated with meltwater input confine most of the primary production to a relatively shallow layer, thereby concentrating microbial cells near the surface. In contrast, the open‐ocean waters of the Drake Passage have a much deeper mixed layer and lower particle loads, allowing light and phytoplankton biomass to extend to greater depths and rely on high nutrient concentrations in the Southern Ocean (Pollard et al. 2006). Overall, microbial cell abundances declined southward, except for photosynthetic nanoeukaryotes (PNE), which increased at the Drake Passage. Higher surface abundances of Cryptophytes in Parry Fjord and Almirantazgo Sound coincided with lower salinity and PNE significantly correlated negatively with density (*R* ^2^ = −0.53) and positively with photosynthetically active radiation (PAR) (*R* ^2^ = 0.52) (Table S2). Across the Garibaldi Fjord, the Beagle Channel and the Drake Passage, we observed a decoupling between Chl‐a concentration and phytoplankton abundance (i.e., low cellular abundances but high Chl‐a) (Figure S3), likely attributed to larger (> 20 μm) phytoplankton.

### 3.2 Distinct Microbial Communities Characterise Fjords and Channels in Southern Patagonia

Across the full dataset (combining pico and nano size fractions; Table S1), Proteobacteria (56.5% ± 19.7%) and Bacteroidota (28.7% ± 15.4%) dominated the bacterial community, mainly represented by Alphaproteobacteria, Bacteroidia and Gammaproteobacteria. The most abundant families were Rhodobacteraceae, Flavobacteriaceae and SAR11 Clade I, the latter showing marked spatial variability from ~5% in Beagle and Garibaldi to ~29% in the Drake Passage (Figure S4A). The eukaryotic plankton community, excluding dinoflagellates to avoid their high relative read abundance that could obscure the patterns of other taxa, was dominated by Haptista (39.4% ± 26%), TSAR (23.8% ± 16.9%) and Archaeplastida (20.8% ± 17.5%), with Phaeocystales, Oligotrichida and Mamiellales as the leading taxa (Figure S4B). Mamiellales, a known photosynthetic picoplanktonic member, declined from ~23% in Almirantazgo and Ballenero/Cockburn to ~2% in Garibaldi–Beagle–Drake, consistent with the reduced PPE abundances (see Figure S3). Finally, dinoflagellate communities were dominated by Syndiniales (28.2% ± 15%). Gymnodiniaceae (26.7% ± 21.6%) and *Gyrodinium* (26.9% ± 20%) were also dominant (Figure S4C).

**FIGURE 2:**
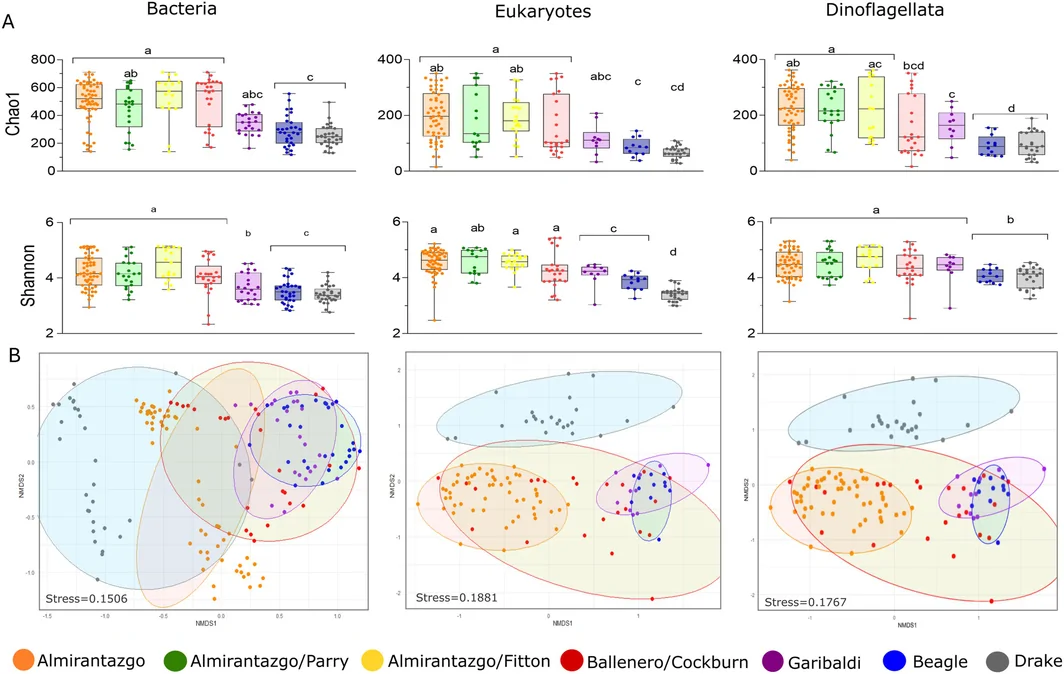
Alpha and beta diversity of bacterial, eukaryotic and dinoflagellate communities. (A) Boxplots show alpha diversity (Chao1 and Shannon indices) across sampling areas. Letters indicate significant differences among areas (*p* < 0.05, Mann–Whitney test). (B) NMDS ordinations based on Bray–Curtis dissimilarities illustrate differences in microbial community composition among areas. Ellipses represent 95% confidence intervals derived from sample score covariance. Stress values for each ordination are shown in the corresponding plot. Points plotted correspond to stations 1–36 to exclude possible temporal variations of the return track samples. Different depths for each station are plotted.

Alpha diversity patterns differed among regions for both bacteria and eukaryotes. Bacterial diversity was highest in the Almirantazgo and Ballenero/Cockburn areas and lowest at the Beagle Channel and Drake Passage. For microbial eukaryotes, richness was lower and more uniform in Garibaldi Fjord, Beagle Channel and Drake, while diversity declined southward (Figure 2A).

Multidimensional scaling based on compositional dissimilarities indicated clear differentiation among fjord and channel and open‐ocean microbial communities (Figure 2B). PERMANOVA confirmed weak but significant regional differences in bacterial and microbial eukaryotes' composition, including dinoflagellates, among the four main areas: Almirantazgo area, Garibaldi Fjord, Ballenero/Cockburn Channels and Beagle Channel (*p* < 0.05) (Table S3). Redundancy analyses further indicated that salinity was the only environmental variable that consistently explained a significant fraction of the variation in both bacterial and microbial eukaryote distributions (Figure S5). However, these analyses are based on observational field data and, while they identify statistically significant associations, do not allow us to infer causality.

The observed spatial structuring of microbial communities is consistent with strong salinity gradients generated by glacier melt, which are associated with shifts in bacterial, archaeal and eukaryotic assemblages in Patagonian fjords and other polar‐influenced systems (González et al. 2013; Gutiérrez et al. 2015; Cuevas et al. 2019; Tamayo‐Leiva et al. 2021).

### 3.3 Core Bacterial Communities Are Widespread and Stable Across Fjords and Channels, Whereas Eukaryotic Core Communities Are Spatially Restricted

**FIGURE 3:**
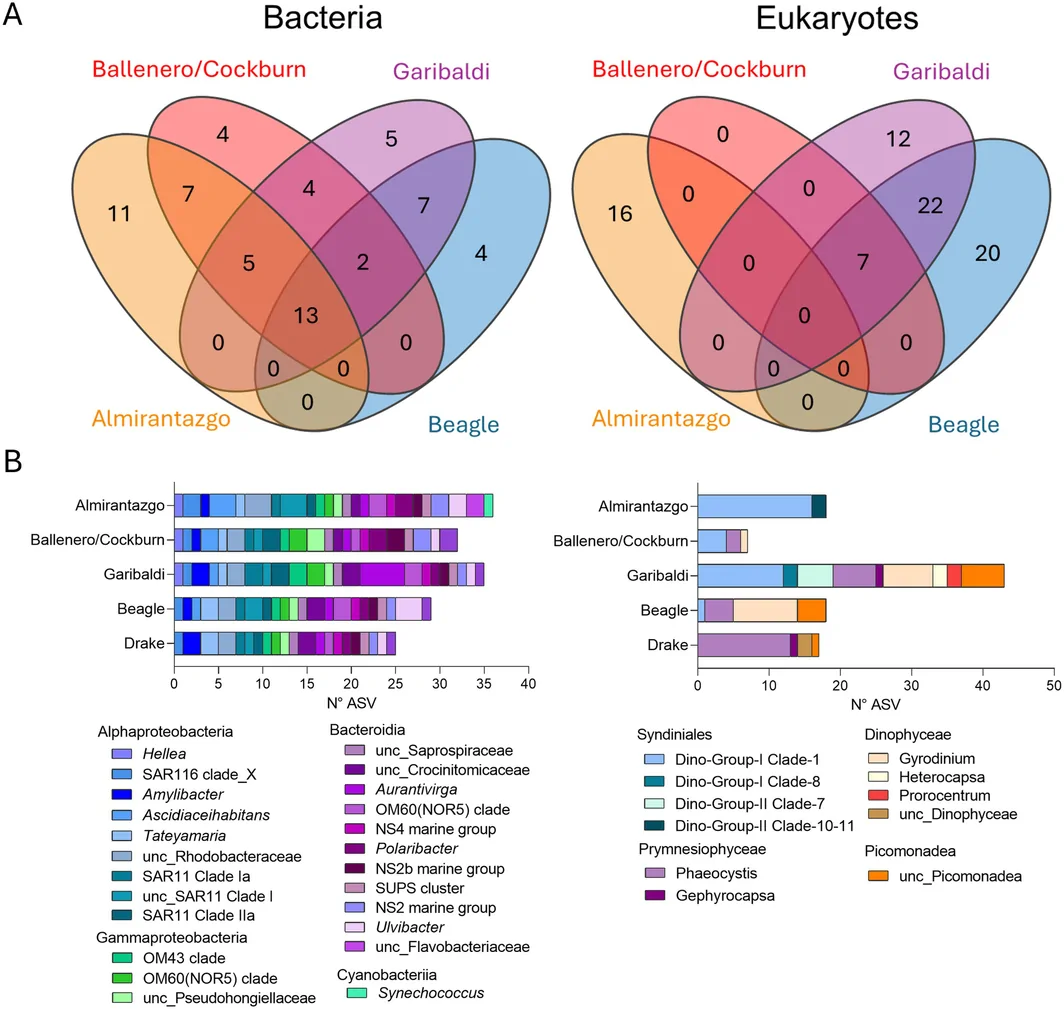
Core bacterial and eukaryotic ASVs among sampling areas. (A) Venn diagrams show the number of core ASVs shared and unique to each area for bacteria (left) and eukaryotes (right). Colours correspond to the main sampling areas: Almirantazgo (yellow), Ballenero/Cockburn (red), Garibaldi (purple) and Beagle (blue). (B) Taxonomic composition of ASV belonging to the core community at genus level for bacteria and microbial eukaryotes. Genera are divided for colour coding at the class level.

We defined the core microbiome as ASVs occurring at > 0.1% relative abundance in at least 80% of samples within a given area, a combined occupancy–abundance approach that selects dominant and persistent taxa while excluding rare, potentially transient lineages (Neu et al. 2021). The analysis identified 62 bacterial ASVs (4.4% of total ASVs > 0.1%) and 77 eukaryotic ASVs (1.8% of total ASVs > 0.1%), exhibiting distinct domain‐specific patterns of ubiquity and exclusivity. Of the 62 bacterial ASVs, 13 were shared by the four areas, 25 were shared by at least two areas and 24 were exclusive to one area. Conversely, of the 77 eukaryotic core ASVs, none was shared by the four areas, 29 were shared between areas and 48 were exclusive to each area, evidencing less ubiquity when compared with the bacterial core (Figure 3A). The bacterial core was dominated by the SAR11 clade, Rhodobacterales and Flavobacterales. Notably, two of the 11 ASVs exclusive to Almirantazgo belonged to Cyanobacteria and Actinobacteria, taxa absent from the other sites, whose cores comprised only Bacteroidia and Proteobacteria (Figure 3B). In eukaryotes, the exclusive ASVs from Almirantazgo and Garibaldi were almost entirely Syndiniales, with the Almirantazgo ASVs showing no overlap with those from other regions. Conversely, the six ASVs exclusive to Beagle Channel were identified as *Phaeocystis* and *Gyrodinium* (Figure 3B), which have been associated with glacier‐ or meltwater‐influenced fjords (Rodríguez‐Marconi et al. 2024).

Core ASVs represented 93% of the bacterial and 88% of the eukaryotic total reads, but only 0.7% and 0.3% of the total ASVs, respectively. This strong dominance of a small set of ASVs highlights that a relatively small core can account for most of the sequencing signal in these fjord systems. Taken together, these results support the use of the core microbiome concept at a regional oceanographic scale, even in free‐living communities that are subject to dispersal among sites and the water column (Mende et al. 2019) and show that bacterial cores are geographically widespread, whereas eukaryotic cores are much more spatially restricted and likely tied to local bloom dynamics.

Taken together, these large‐scale spatial patterns indicate that microbial communities vary markedly across the fjord–channel–open ocean continuum at a regional scale, implying that local ecosystem functioning is likely shaped by distinct, area‐specific microbiomes.

### 3.4 Functional and Trophic Differentiation Across Fjord and Channel Communities

Meltwater influence shapes microbial metabolic potential along the freshwater‐marine continuum within Greenland fjords, enriching diverse pathways that reflect adaptive functional plasticity and modulate organic matter supply (Balmonte et al. 2021).

**FIGURE 4:**
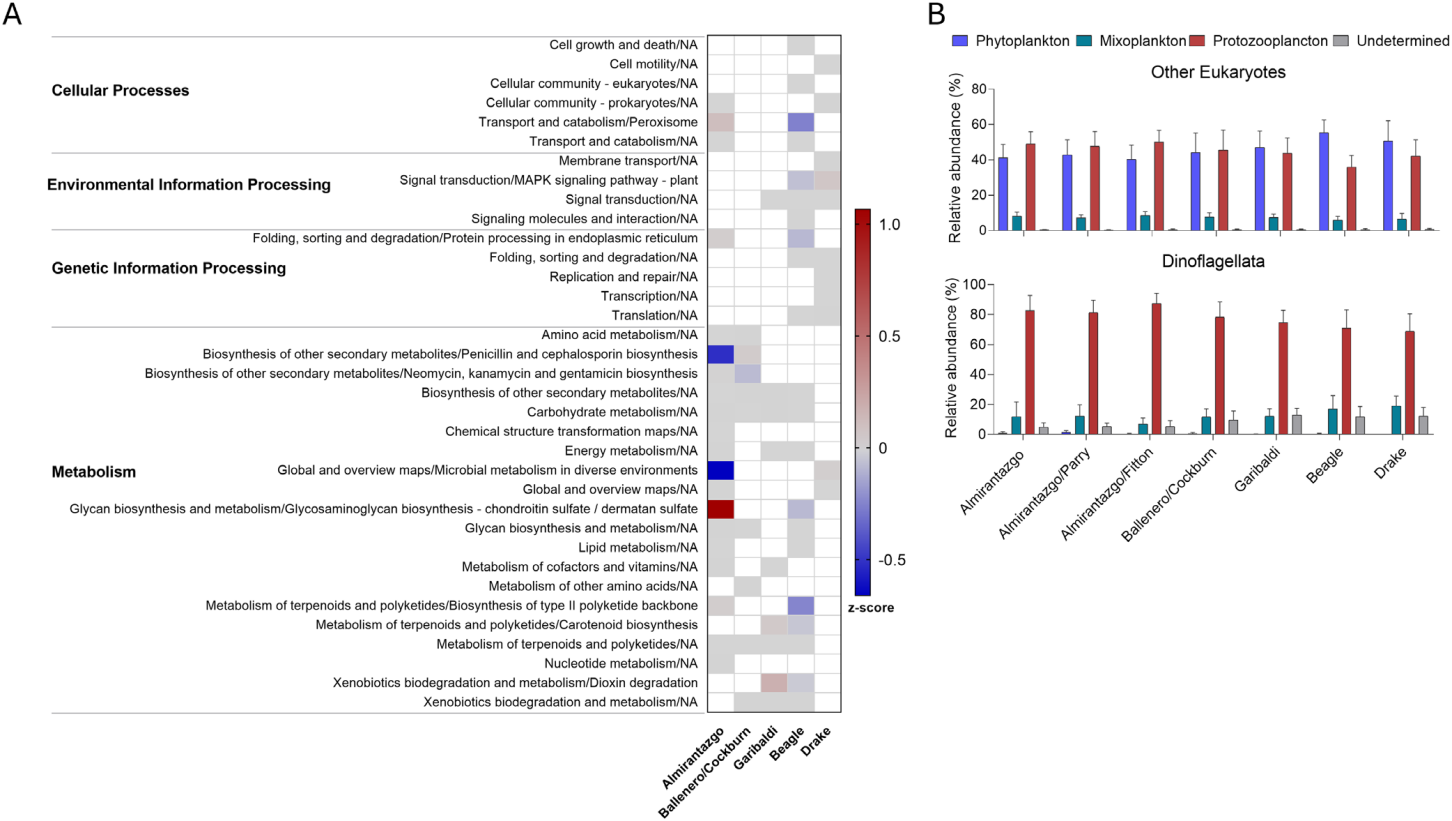
Functional profiles and trophic composition of microbial eukaryotes across Patagonian fjord systems. (A) Differential abundance (*z*‐scores) of predicted KEGG functional categories for bacterial communities grouped into major pathways in each main area of the study. Warmer colours indicate functions enriched at a given site, whereas cooler colours indicate reduced representation. (B) Relative sequence abundance of major trophic categories of microeukaryotes in surface waters of each study area, shown separately for ‘Other Eukaryotes’ (top) and Dinoflagellata (bottom). Bars represent the proportion of phytoplankton (blue), mixoplankton (green), protozooplankton (red) and undetermined trophic modes (grey) for each main area, with error bars indicating standard deviation among samples.

Here, functional predictions based on 16S rRNA genes revealed consistency in most bacterial KEGG pathways across areas, though a subset of metabolic pathways differed (Figure 4A). Specifically, in the Almirantazgo area, biosynthesis of glycosaminoglycan‐like molecules was enriched, suggesting a community more strongly geared towards exopolysaccharide (EPS) synthesis, a capacity linked to biofilm integrity, carbon turnover modulation and persistence under fluctuating salinity and nutrient regime (Decho and Gutierrez 2017). Conversely, pathways for penicillin and cephalosporin biosynthesis were reduced, suggesting diminished investment in antibiotic‐mediated competition and the broader signalling roles these compounds play in biofilm dynamics and eukaryotic interactions (Linares et al. 2006; Cordero et al. 2012; Wietz et al. 2013; Liu et al. 2025).

Because of the limitation associated with inferring functional traits from 18S rRNA gene sequences (e.g., Stoeck et al. 2010; Keeling and Del Campo 2017), we classified eukaryotic function based on their trophic profiles as phytoplankton, mixoplankton, or protozooplankton according to published information on their nutritional mode (Schneider et al. 2020 and references therein). The general trophic composition of microbial eukaryotes showed no significant differences between areas. Among non‐dinoflagellates, phytoplankton and protozooplankton taxa contributed almost equally (45% ± 5.1% and 44.8% ± 4.5%, respectively), whereas mixoplankton made up only a minor fraction (7.3% ± 0.8%). In contrast, within dinoflagellates, protozooplankton dominated (77.7% ± 6.1%), mixoplankton were intermediate (12.9% ± 3.6%) and phytoplanktonic dinoflagellates were almost absent (0.45% ± 0.36%) (Figure 4B). This substantial heterotrophic component within the eukaryotic plankton agrees with observations near the heads of the Patagonian fjords between 47° and 50° S, where trophic fluxes were dominated by heterotrophic nanoflagellates (González et al. 2013).

Taken together, the observed potential functional composition in our study likely reflects a post‐bloom situation in which abundant bacteria may be interacting with microbial eukaryotes in more than one way. One is associated with particles through EPS production and potentially being modulated by antibiotic production. On the other hand, bacteria can be stimulated by elevated DOM from preceding phytoplankton blooms and can also be efficiently grazed by heterotrophic protists. Experiments in Arctic and sub‐Arctic systems further show that additions of phytoplankton‐derived or other labile DOM stimulate bacterial growth (Kim, Han, et al. 2022; Kim, Kim, et al. 2022). In Chilean Patagonian fjords, inputs of salmon‐food‐derived and glacially influenced DOM similarly promote bacterial activity and reshape bacterioplankton community structure (Montero et al. 2022).

### 3.5 Contrasting Glacier Types and Study Areas Display Different Interactions Between Bacteria and Microbial Eukaryotes

We focused on microbial interactions between bacteria and microbial eukaryotes by contrasting interdomain co‐occurrence networks between Parry and Garibaldi Fjords (water‐terminating glaciers) and Fitton Bay (land‐terminating glacier). Network‐level topological properties were distinguishable across these study areas (Table S4), but all exhibited sparse connectivity, typical of biological networks (Busiello et al. 2017). Fitton Bay had the highest number of nodes (i.e., ASVs), edges (i.e., significant abundance correlations) and hub nodes (i.e., highly connected bacteria and microbial eukaryotes), with more than three times the average number of neighbours compared with the water‐terminating glaciers and exhibited mild heterogeneity. Parry Fjord showed few dominant hubs (high heterogeneity), while Garibaldi Fjord had more uniform connections (low heterogeneity). We found significant differences among the three networks for almost all node‐level topological properties (Tables S5 and S6), except for the topological coefficient. The abundance and number of positive interactions were two properties that were significantly similar only between the water‐terminating glacier zones (Parry and Garibaldi fjords).

**FIGURE 5:**
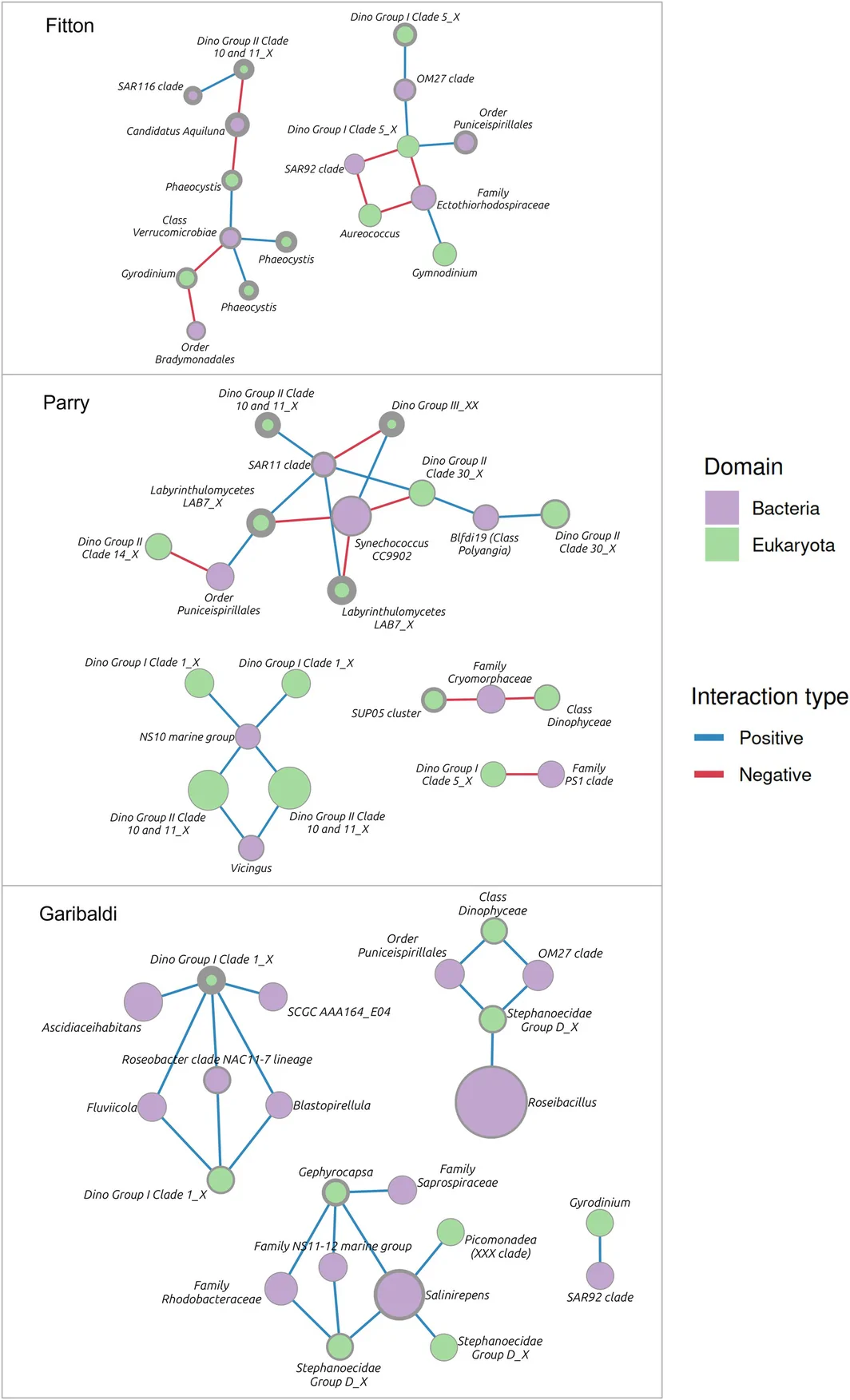
Bipartite co‐occurrence networks of bacteria and microbial eukaryotes with the highest betweenness centrality, from contrasting glacier types. Nodes represent ASVs and edges represent significant abundance correlations between bacterial and eukaryotic ASVs, with Pearson's *r* and Spearman's *ρ* > 0.8 and *p* < 0.01. Edge colour indicates direct (positive) or inverse (negative) correlations (interactions) between pairs of nodes. Node size symbolises ASV abundance and border width symbolises the node degree (i.e., the number of connections per node), both scaled within networks but not precisely comparable across them.

We analysed the most important interactions between bacteria and microbial eukaryotes across zones by identifying the nodes with the highest betweenness centrality (Figure 5). These are nodes that appear most frequently along the shortest paths between node pairs, also termed ‘gatekeepers’ and correspond to microorganisms that control network interactions and stability (Dai et al. 2019; Kajihara and Hynson 2024; Lee et al. 2022). Among these microbial gatekeepers, several members of the Syndiniales (Dino Group I and II, III) stood out in Parry Fjord. Clade‐1 species (Dino Group I) represent important parasites that interacted positively with several bacterial gatekeepers in Parry and Garibaldi fjords (water‐terminating glaciers). Meanwhile, clade‐5 species (Dino Group I) and clade‐10/11 species (Dino Group II) were important members that interacted positively and negatively with several bacterial gatekeepers in Parry Fjord and Fitton Bay (both in the Almirantazgo area). Garibaldi Fjord showed only positive interactions among all microbial gatekeepers.

Fitton Bay (land‐terminating glacier) was characterised by a greater evenness of microbial gatekeepers and the centrality of hubs of *Phaeocystis*, Dino Group II clade 10/11, *Gyrodinium*, Candidatus *Aquiluna*, class Verrucomicrobiae and SAR 116 (class Alphaproteobacteria). Parry Fjord presented a smaller set of dominant hub gatekeepers, notably of Labyrinthulomycetes (Stramenopiles) and Dino Group III, both interacting with *Synechococcus*; whereas Garibaldi Fjord was dominated by a single hub gatekeeper of clade‐1 Dino Group I. Betweenness and degree centralities have been commonly used to identify keystone taxa in environmental and host‐associated microbial communities (Tipton et al. 2018; Wall et al. 2020; Zamkovaya et al. 2021). In agreement, hub and gatekeeper microorganisms identified here (some also present in core microbiomes, as *Synechococcus* and Dino Group I), representing keystone taxa, may have critical ecological importance in maintaining community structure and functionality, with their removal affecting community connectivity in a cascading manner (Berry and Widder 2014; Kajihara and Hynson 2024).

Overall, the three fjords differ markedly in the identity and distribution of microbial hubs and gatekeepers, indicating contrasting modes of community interactions. Identifying these putative keystone taxa provides specific targets to monitor how changes in glacier type and freshwater input may rewire bacteria–eukaryote networks in Patagonian fjords.

Although interdomain network studies are still rare, recent work in the Arctic Svalbard fjords using amplicon sequencing showed network links between Prasinophyceae or Dinophyceae and Actinobacteria, reflecting potential exchanges of phytoplankton‐derived organic matter (Han et al. 2024). In this context, the central positions of Dino Groups I–III, Labyrinthulomycetes, *Phaeocystis*, *Candidatus* Aquiluna and SAR116, among others, suggest that they act as interdomain connectors, potentially mediating the transfer of organic matter between phytoplankton, heterotrophic and mixotrophic protists and bacterioplankton. Connecting with our functional inferences, increased EPS biosynthetic capacities in the Almirantazgo area (including Fitton Bay and Parry Fjord) (see Figure 4) suggest community aggregation trends, consistent with these strong inter‐domain co‐occurrences.

### 3.6 Short‐Scale Temporal Dynamics in Bacterial and Microbial Eukaryotic Communities at Garibaldi Fjord Are Reflected in Pronounced Taxonomic and Functional Shifts

Taking advantage of the 10‐day interval between the outbound and return legs of the cruise across the Garibaldi Fjord, we examined short‐term changes in the taxonomic and functional diversity and composition of bacterial and microbial eukaryote communities in this specific area. Nitrate levels were significantly lower in the outbound leg than in the return. Cell abundances of PPE and PNE were significantly lower during the outbound leg (Kolmogorov–Smirnov p: 7e−7 and p: 0.016, respectively). No difference was observed in average bacterial cell numbers and Chl‐a concentration between the outbound and return legs (Figure S6).

**FIGURE 6:**
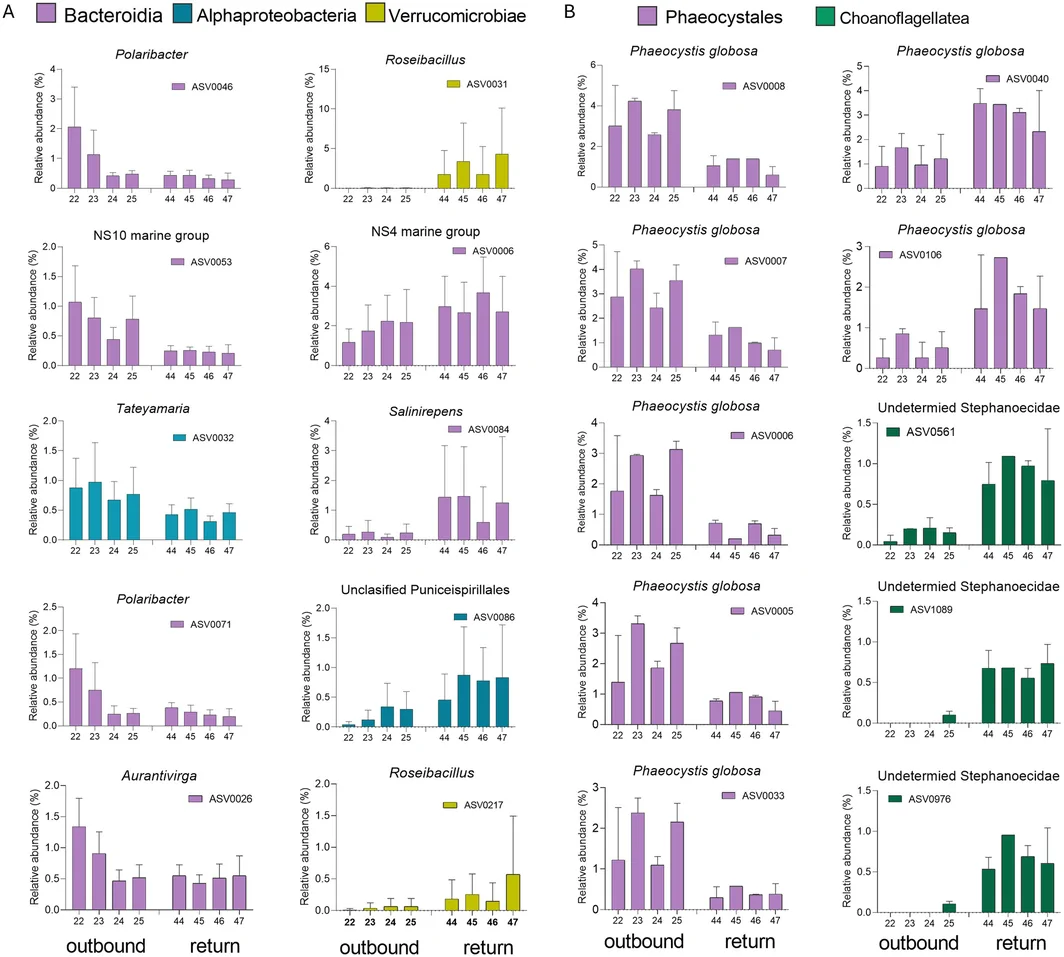
Temporal changes over the 10‐day interval in the relative abundance of selected bacterial (A) and microbial eukaryote (B) ASVs during the outbound and return legs in the Garibaldi Fjord. Bars represent mean relative abundance (%) for each sampling station (*x*‐axis) and error bars indicate standard deviation among samples. Left and right panels for bacteria and microeukaryotes show ASVs that significantly decreased or increased, respectively, between the two tracks.

LDA identified a decrease in abundance of two *Polaribacter* ASVs, one NS10 marine group, one *Tateyamaria* and one *Aurantivirga* between the outbound and return legs. Conversely, two *Roseibacillus* ASVs, one *Salinirepens*, one NS4 marine group and one Puniceispirillales increased in relative abundance after the 10‐day period (Figure 6A). Among eukaryotes, five ASVs declined, including *Phaeocystis globosa* and an unknown member of Stephanoecidae, while two other *P. globosa* ASVs increased in relative abundance (Figure 6B).

In addition to varying significantly within this 10‐day period, ASVs from the order Puniceispirillales, *Roseibacillus* and *Salinirepens* within Bacteria and the Group Stephanoecidae within eukaryotes (Figures 5 and 6) also appeared within the inter‐domain co‐occurrence network from Garibaldi Fjord (see Figure 5), suggesting that rapid shifts in microbial abundance may be driven not only by environmental factors but also by biological interactions. We compared members of the core microbiome with highly connected taxa in network analyses. Interestingly, ASV assigned to the group Puniceispirillales was also found to be part of the core at Garibaldi Fjord. This group may play an important ecological role given its recurrence and strong inter‐domain interactions.

Interestingly, bacterial functional profiling revealed marked differences in pathway representation between the two tracks. While the outbound leg was characterised by increased activity in signalling and stress‐response pathways, the return leg showed a greater representation of biosynthetic and energy metabolism pathways (Figure S7A), suggesting a possible shift from a stress‐response state to a more metabolically stable and productive phase. Within eukaryotes, excluding dinoflagellates, the second track had a higher relative abundance of protozooplankton (Figure S7B). Previous studies on Patagonian Fjords showed that although abiotic factors directly or indirectly affect particular taxa within the microbial assemblages, biological interactions can permeate the entire community structure (Tamayo‐Leiva et al. 2021). Our results further highlight the fundamental role of bacteria‐eukaryote interactions, as well as the short timing of these ecological and metabolic processes in shaping summer microbial communities in Patagonian fjord ecosystems.

Unfortunately, our study focuses on a single summer period, so we still lack information on how these microbial cores and networks change across seasons, years and extreme melt events. We also lack direct measurements of metabolic functions or process rates, which limits how far we can interpret the inferred functional profiles. Future work combining high‐resolution time series, metagenomics, metatranscriptomics and experimental measurements of bacterial production, respiration, grazing, etc. will be essential to quantify how changes in glacier stability and freshwater input propagate into changes in the functioning of Patagonian fjord ecosystems.

## 4 Conclusions

Together, our results show that bacterial cores are geographically widespread, whereas eukaryotic cores are much more spatially restricted. Our findings also highlight a subset of microorganisms that function as keystone inter‐domain species that are not necessarily the same taxa across geographically similar environments. More broadly, this study illustrates the large‐scale extent of variation in microbial communities across Patagonian fjords and channels and shows how such variation is closely linked to the functioning of these environments. Given the rapid retreat of Patagonian glaciers, identifying both these persistent and keystone microorganisms provides a baseline for anticipating how fjord microbiomes will respond to warming and altered runoff regimes. Knowing, understanding and valuing the role that microorganisms, both bacteria and microbial eukaryotes, play in these marine environments under the effects of global change should be key to protection, conservation and management plans.

## Author Contributions

**Nicole Trefault:** conceptualization, investigation, funding acquisition, writing – original draft, writing – review and editing, project administration, resources, supervision. **Susana Rodríguez‐Marconi:** investigation, conceptualization, writing – original draft, methodology, visualization, writing – review and editing, formal analysis, data curation. **María Fernanda Manrique‐de‐la‐Cuba:** investigation, methodology, visualization, writing – review and editing, formal analysis. **Patricio Flores‐Herrera:** methodology, writing – review and editing. **Clara Natalia Rodríguez‐Flórez:** methodology, writing – review and editing. **Bernd Krock:** funding acquisition, writing – review and editing, supervision.

## Funding

This work was supported by the Bundesministerium für Bildung und Forschung (LAT16STRUC‐039), the Horizon 2020 Framework Programme (872690) and the Agencia Nacional de Investigación y Desarrollo (Fondecyt No. 1230758).

## Conflicts of Interest

The authors declare no conflicts of interest.

## Supporting information


**Table S1:** Sampling station locations and depths (m) for each analytical category measured (Chlorophyll‐a, nutrients, cell abundance by flow cytometry and 16S/18S rRNA tag sequencing of pico‐ and nano‐plankton size fractions) during the FjordFlux cruise. Numbers indicate the sampling depths in metres for each sample type. The number of columns for each sample type depends on the number of discrete depths from which samples were taken.
**Table S2:** Correlation of oceanographic variables and cell abundance data. Spearman *R* ^2^ (below diagonal) and *p*‐value (above diagonal) are displayed among log‐transformed oceanographic parameters and cell abundances. Only significant values are shown.
**Table S3:** Pairwise PERMANOVA results associated with NMDA analysis.
**Table S4:** Network‐level topological properties.
**Table S5:** Statistical comparisons between node‐level topological properties of networks. ****p < 0.0001, ***p < 0.001, **p < 0.01, *p < 0.05.
**Table S6:** Node‐level topological properties of networks.

**Figure S1:** Vertical profiles of physicochemical conditions at Parry Bay using CTD measurements in each station.
**Figure S2:** Physicochemical surface conditions. Surface CTD profiles from each station are shown for the fjord and channel region (A) and the Drake Passage (B).
**Figure S3:** Cell abundances, nutrients and chlorophyll‐a distribution across sampling areas. Boxplots represent cell abundances of bacteria, picoeukaryotes (PPE) and nanoeukaryotes (NPE) (integrated within the photic zone), surface concentrations of nitrate (NO_3_^−^), nitrite (NO_2_^−^), ammonium (NH_4_^+^), phosphate (PO_4_^3−^) and silicic acid (Si) and chlorophyll‐a concentrations at each area (mostly at 10 m depth, see Table S1 for details), showing mean values and standard deviations. Letters indicate significant differences among areas (*p* < 0.05, Mann–Whitney test).
**Figure S4:** Community composition across Patagonian fjords, channels and the Drake Passage. Stacked bar plots show the average relative abundance of (A) bacterial families, (B) microbial eukaryotes excluding dinoflagellates and (C) dinoflagellates, within the upper 30 m of the water column at each station. Taxa are grouped by their corresponding two higher taxonomic levels as indicated in the legends; taxa contributing < 1% or 2% of total sequences are pooled into ‘Others’.
**Figure S5:** Redundancy analysis (RDA) of microbial community composition constrained by environmental variables. Each point represents average community composition within the first 30 m of the water column, coloured by sampling area. Arrows represent environmental variables that significantly correlate with community composition, with arrow length proportional to the strength of the correlation. Adjusted *R* ^2^ indicates the proportion of community variation explained by the selected environmental variables.
**Figure S6:** Short‐term variability in nutrients and microbial abundances at Garibaldi Fjord. Bar plots show surface concentrations of nitrate (NO_3_^−^), nitrite (NO_2_^−^), ammonium (NH_4_^+^), phosphate (PO_4_^3−^) and silicic acid (Si), together with chlorophyll a (Chl‐a) and abundances of bacteria, picoeukaryotes (PPE) and nanoeukaryotes (NPE) at stations 22–25 and 44–47 during the outbound and return legs of the cruise. Error bars indicate standard deviation among replicate samples where available.
**Figure S7:** (A) Heatmap showing the relative representation of KEGG Level 3 pathways between the Outbound and Return phases of the cruise transect at Garibaldi Fjord. *Z*‐scores represent normalised pathway abundances across samples, where red and blue indicate positive and negative deviations from the mean, respectively. (B) Trophic classification. Bar plots represent the average and SD of reads assigned to the three functional groups in the first (Outbound) and second (Return) track at Garibaldi Fjord. Undetermined represent genera or species that could not be found in the database and ASV that were assigned to a higher taxonomic level. Characters show significant differences.

## Data Availability

The data that support the findings of this study are openly available in NCBI Short Read Archive at https://www.ncbi.nlm.nih.gov/sra, reference number PRJNA1394180 for 16S rRNA and PRJNA1395643 for 18S rRNA.
