# Supplementary material for: Stable Bacterial Cores and Patchy Eukaryotic Communities Shape the Summer Microbiomes of Patagonian Fjord Ecosystems Across Spatial, Functional and Temporal Gradients
Source: Environ Microbiol Rep. 2026 Sep 8;18(5):e70412. doi: 10.1111/1758-2229.70412 (PMC13554426; doi:10.1111/1758-2229.70412)
Supplement: Supplementary file 1 [file EMI4-18-e70412-s002.docx]

**Supporting Information**

**Stable bacterial cores and patchy eukaryotic communities shape the summer microbiomes of Patagonian fjord ecosystems across spatial, functional, and temporal gradients**

Table S1. Sampling station locations and depths (m) for each analytical category measured (Chlorophyll‑a, nutrients, cell abundance by flow cytometry, and 16S/18S rRNA tag sequencing of pico‑ and nano‑plankton size fractions) during the FjordFlux cruise. Numbers indicate the sampling depths in meters for each sample type. The number of columns for each sample type depends on the number of discrete depths from which samples were taken.

Table S2. Correlation of oceanographic variables and cell abundance data. Spearman R-squared (below diagonal) and p-value (above diagonal) are displayed among log-transformed oceanographic parameters and cell abundances. Only significant values are shown.

Table S3. Pairwise PERMANOVA results associated with NMDA analysis.

Table S4. Network-level topological properties.

Table S5. Statistical comparisons between node-level topological properties of networks. ****: p<0.0001, ***: p<0.001, **: p<0.01, *: p<0.05

Table S6. Node-level topological properties of networks.

Figure S1. Vertical profiles of physicochemical conditions at Parry Bay using CTD measurements in each station.

A

B

Figure S2. Physicochemical surface conditions. Surface CTD profiles from each station are shown for the fjord and channel region (A) and the Drake Passage (B).

Figure S3. Cell abundances, nutrients, and chlorophyll-a distribution across sampling areas. Boxplots represent cell abundances of bacteria, picoeukaryotes (PPE), and nanoeukaryotes (NPE) (integrated within the photic zone), surface concentrations of nitrate (NO₃⁻), nitrite (NO₂⁻), ammonium (NH₄⁺), phosphate (PO₄³⁻), and silicic acid (Si), and chlorophyll-a concentrations at each area (mostly at 10 m depth, see table S1 for details), showing mean values and standard deviations. Letters indicate significant differences among areas (p < 0.05, Mann–Whitney test).

Figure S4. Community composition across Patagonian fjords, channels, and the Drake Passage. Stacked bar plots show the average relative abundance of (A) bacterial families, (B) microbial eukaryotes excluding dinoflagellates, and (C) dinoflagellates, within the upper 30 m of the water column at each station. Taxa are grouped by their corresponding two higher taxonomic levels as indicated in the legends; taxa contributing <1% or 2% of total sequences are pooled into “Others.”

Figure S5. Redundancy analysis (RDA) of microbial community composition constrained by environmental variables. Each point represents average community composition within the first 30m of the water column, colored by sampling area. Arrows represent environmental variables that significantly correlate with community composition, with arrow length proportional to the strength of the correlation. Adjusted R² indicates the proportion of community variation explained by the selected environmental variables.

Figure S6. Short‑term variability in nutrients and microbial abundances at Garibaldi Fjord. Bar plots show surface concentrations of nitrate (NO₃⁻), nitrite (NO₂⁻), ammonium (NH₄⁺), phosphate (PO₄³⁻), and silicic acid (Si), together with chlorophyll a (Chl‑a) and abundances of bacteria, picoeukaryotes (PPE), and nanoeukaryotes (NPE) at stations 22–25 and 44–47 during the outbound and return legs of the cruise. Error bars indicate standard deviation among replicate samples where available.

Figure S7. A) Heatmap showing the relative representation of KEGG Level 3 pathways between the Outbound and Return phases of the cruise transect at Garibaldi Fjord. Z-scores represent normalized pathway abundances across samples, where red and blue indicate positive and negative deviations from the mean, respectively. B) Trophic classification. Bar plots represent the average and SD of reads assigned to the three functional groups in the first (Outbound) and second (Return) track at Garibaldi Fjord. Undetermined represent genera or species that could not be found in the database, and ASV that were assigned to a higher taxonomic level. Characters show significant differences.
